# P-Rex1 Controls Sphingosine 1-Phosphate Receptor Signalling, Morphology, and Cell-Cycle Progression in Neuronal Cells

**DOI:** 10.3390/cells10092474

**Published:** 2021-09-18

**Authors:** Elizabeth Hampson, Elpida Tsonou, Martin J. Baker, David C. Hornigold, Roderick E. Hubbard, Andrew Massey, Heidi C. E. Welch

**Affiliations:** 1 Signalling Programme, The Babraham Institute, Babraham Research Campus, Cambridge CB22 3AT, UK; Elizabeth.Hampson@babraham.ac.uk (E.H.); elpida.tsonou1@astrazeneca.com (E.T.); martin.baker@manchester.ac.uk (M.J.B.); 2 Bioscience Metabolism, Research and Early Development, Cardiovascular, Renal and Metabolism (CVRM), BioPharmaceuticals R&D, AstraZeneca, Cambridge CB21 6GH, UK; David.Hornigold@astrazeneca.com; 3 Vernalis (R&D) Ltd., Cambridge CB21 6GB, UK; r.hubbard@vernalis.com (R.E.H.); a.massey@vernalis.com (A.M.)

**Keywords:** P-Rex1, guanine-nucleotide exchange factor, GEF, Rac1, Rac3, Rho GTPase, G protein-coupled receptor (GPCR), neuronal signalling, cell morphology, cell-cycle progression, cell proliferation

## Abstract

P-Rex1 is a guanine-nucleotide exchange factor (GEF) that activates Rac-type small G proteins in response to the stimulation of a range of receptors, particularly G protein-coupled receptors (GPCRs), to control cytoskeletal dynamics and other Rac-dependent cell responses. P-Rex1 is mainly expressed in leukocytes and neurons. Whereas its roles in leukocytes have been studied extensively, relatively little is known about its functions in neurons. Here, we used CRISPR/Cas9-mediated P-Rex1 deficiency in neuronal PC12 cells that stably overexpress the GPCR S1PR1, a receptor for sphingosine 1-phosphate (S1P), to investigate the role of P-Rex1 in neuronal GPCR signalling and cell responses. We show that P-Rex1 is required for the S1P-stimulated activation of Rac1 and Akt, basal Rac3 activity, and constitutive cAMP production in PC12-S1PR1 cells. The constitutive cAMP production was not due to increased expression levels of major neuronal adenylyl cyclases, suggesting that P-Rex1 may regulate adenylyl cyclase activity. P-Rex1 was required for maintenance of neurite protrusions and spreading in S1P-stimulated PC12-S1PR1 cells, as well as for cell-cycle progression and proliferation. In summary, we identified novel functional roles of P-Rex1 in neuronal Rac, Akt and cAMP signalling, as well as in neuronal cell-cycle progression and proliferation.

## 1. Introduction

P-Rex1 is a guanine-nucleotide exchange factor (GEF) for Rac-type small guanine nucleotide-binding proteins (GTPases; Rac1, Rac2, Rac3, and RhoG) [1,2,3]. As a typical activator of Rac proteins, P-Rex1 controls cell morphology, adhesion, and chemotaxis through cytoskeletal dynamics, as well as reactive oxygen species (ROS) production, gene expression, and cell survival [2,3]. P-Rex1 is primarily expressed in leukocytes and in the nervous system, but is found at lower levels in many cell types [1,2,3]. P-Rex1 function has mostly been studied in leukocytes and platelets, where this GEF is important for a range of proinflammatory and immune functions, including adhesion, migration, and ROS production [1,4,5,6,7,8,9,10,11,12,13,14]. P-Rex1 is also known for controlling the proliferation and migration of melanocytes during development [15,16], the morphology and migration of fibroblasts [17,18,19], the migration, secretory, and barrier functions of endothelial cells under proangiogenic or inflammatory conditions [20,21,22], and the glucose uptake and thermogenic capacities of adipocytes [23,24]. In addition, P-Rex1 levels are commonly deregulated in many types of cancer, including melanoma, breast, and prostate cancer, promoting tumour growth or metastasis [2,3,15,25,26,27].

P-Rex1 is highly and widely expressed throughout the nervous system [28,29]. Interestingly, single-nucleotide and copy-number variations of the *PREX1* gene are associated with autism spectrum disorders in humans [30]. Perhaps surprisingly given its expression profile, P-Rex1-deficient mice show no overt defects in brain anatomy, but they do exhibit neurological and behavioural phenotypes [29,30,31,32]. They show deficits in social recognition, reversal learning, and fear extinction, which are classified as autism-like behaviours, thus recapitulating the human condition [30,32]. Social recognition and behavioural flexibility depend on hippocampal function. Acute knockdown of P-Rex1 in the hippocampal CA1 region of wild-type mice induced similar autism-like behaviours to those seen in P-Rex1-deficient mice, suggesting that hippocampal P-Rex1 is responsible for this phenotype [30]. Accordingly, long-term depression was impaired in the hippocampal CA1 region of P-Rex1-deficient mice, associated with impaired internalisation of the receptor GluR2. Importantly, this synaptic plasticity defect, as well as the social recognition and behavioural flexibility impairments, could be rescued by the re-expression of P-Rex1 in pyramidal neurons of the hippocampal CA1 region, but not by a catalytically inactive mutant of P-Rex1 [30]. P-Rex1 is also highly expressed in the cerebellum. Yet, motor coordination, which is under cerebellar control, is normal in P-Rex1-deficient mice, and these mice also exhibit normal cerebellar morphology and synaptic plasticity [29]. Nevertheless, P-Rex1 does contribute to cerebellar plasticity and function, as mice deficient in both P-Rex1 and its homolog P-Rex2 show a greater impairment in the long-term potentiation of cerebellar Purkinje neurons and in motor coordination than mice deficient in P-Rex2 alone [29,33]. Overexpression of P-Rex1 in the medial prefrontal cortex of mice resulted in abnormal neuronal polarity, migration, and dendritic spine morphology, and these mice displayed psychosis-related behaviours [34]. Furthermore, overexpression of a dominant-negative mutant of P-Rex1 lacking the catalytic DH domain was shown to block neuronal migration in the developing cerebral cortex [28,35]. Together, these studies showed that correct levels of P-Rex1 expression and of catalytic Rac-GEF activity within the brain are important for neuronal morphology and plasticity, with wide-ranging consequences for mental wellbeing and behaviours.

Given the importance of neuronal P-Rex1 for the whole organism, relatively little is known about its signalling roles in neurons at the cellular level. Two studies investigated this previously in neuronal PC12 cells, with seemingly opposing conclusions. The first showed that overexpression of P-Rex1 increases nerve growth factor (NGF)-stimulated Rac1 activity, lamellipodia formation, membrane ruffling, spreading, and migration, whereas downregulation of P-Rex1 reduced migration [28]. The second reported that overexpression of P-Rex1 blocks NGF-stimulated neurite outgrowth while activating Rac3, not Rac1, whereas downregulation of P-Rex1 had the opposite effects [36]. This apparent discrepancy and any signalling conditions that may alternately promote P-Rex1 Rac1 or Rac3 GEF activity require further investigation. This is relevant because Rac1 and Rac3 GTPase isoforms have opposing effects on neuronal morphology, with Rac1 inducing spreading and Rac3 inducing contraction of the actin cytoskeleton [37].

P-Rex1 is a Dbl-type Rac-GEF with multiple domains that serve to regulate its activity and subcellular localisation [2,3]. The GEF activity of P-Rex1 is directly activated by the Gβγ subunits of heterotrimeric G proteins downstream of G protein-coupled receptors (GPCRs). In addition, P-Rex1 is also directly activated by the lipid second messenger phosphatidylinositol-3,4,5-trisphosphate (PIP_3_), which is generated by phosphoinositide 3-kinase (PI3K) activity downstream of multiple types of membrane receptors. Gβγ and PIP_3_ can both independently and synergistically activate P-Rex1 [1]. In addition, P-Rex1 activity can be positively and negatively regulated through serine/threonine phosphorylation by protein kinases and phosphatases [38,39,40,41]. Under basal conditions, P-Rex1 is mainly cytosolic, but Gβγ and PIP_3_ [42,43], or the GPCR adaptor protein Norbin [44], induces membrane translocation to bring the GEF into proximity of its substrate Rac.

A particularly complex interplay exists between the P-Rex1 and cAMP-dependent kinase (PKA) signalling pathways. PKA inhibits P-Rex1 Rac-GEF activity [39]. The cAMP-binding domain of the regulatory PKA subunits binds the PDZ domains of P-Rex1, while the catalytic PKA subunits phosphorylate P-Rex1 on S436 to induce an inhibitory intramolecular conformation that prevents activation by Gβγ and PIP_3_ [45,46]. In turn, P-Rex1 can stimulate the plasma membrane translocation of PKA upon stimulation of Gi-coupled GPCRs, with currently unknown functional consequences for PKA signalling [46]. It was recently shown that PKA not only inhibits P-Rex1, but can also activate the Rac-GEF upon stimulation of Gs-coupled GPCRs, independently of its kinase activity [47]. When cAMP produced in response to GPCR stimulation binds to the regulatory PKA subunits, they dissociate from the catalytic subunits, allowing the regulatory subunit to bind P-Rex1 and stimulate its Rac-GEF activity. Different cellular pools of P-Rex1 were shown to be activated by the regulatory subunits and inhibited by the catalytic subunits of PKA. Overall, the catalytic subunits seem to have the predominant effect of inhibiting P-Rex1, whereas the regulatory subunits fine-tune this [47].

To address the relative paucity of information on the functional roles of P-Rex1 in neuronal cells, particularly in the context of GPCR signalling, we report here our investigation of the constitutive and GPCR-stimulated roles of P-Rex1 in the signalling pathways and responses of neuronal PC12 cells. Signalling through the sphingolipid sphingosine 1-phosphate (S1P) is essential for normal neuronal function, with its deregulation resulting in neurodegenerative or neuroinflammatory disease [48]. S1PR1, which is Gα_i_-coupled, is one of five receptors for S1P. We chose to focus our P-Rex1 study on signalling through S1PR1, as this is the most commonly expressed S1P receptor in the brain [49]. The receptor is known to be required for the proliferation, migration, and differentiation of neuronal stem cells [50,51]. Furthermore, P-Rex1 has previously been shown to interact with S1PR1 and to mediate the S1P-stimulated migration of endothelial cells [52].

## 2. Materials and Methods

### 2.1. Cell Culture

PC12 (rat adrenal gland phaeochromocytoma) cells (a kind gift from Dr. Llewelyn Roderick, Babraham Institute, Cambridge, UK) were cultured in a humidified incubator at 37 °C with 5% CO_2_ and used for experiments at between 1 and 12 weeks in culture. Aliquots of cells were stored in liquid nitrogen. Cells were grown in vented tissue culture flasks (Nunc) coated with poly-D-lysine (PDL), in Dulbecco’s modified Eagle’s medium (DMEM) (Gibco, Loughborough, UK, 41965-039) supplemented with 10% horse serum, 5% foetal bovine serum, 1× glutamine, 1 U·mL^−1^ penicillin, and 1 U·mL^−1^ streptomycin (Gibco, 15140-122). Transfections were carried out using JetPei DNA transfection reagent (Polyplus, Illkirch France, 101-10N) according to the manufacturer’s instructions. PC12 cells that stably express C-terminally GFP-tagged S1PR1 (PC12-S1PR1 cells) were generated by transfecting parental PC12 cells with pCDNA-3.1 neo S1PR1-GFP (a kind gift from Prof. Timothy Hla, Weill Medical College, Cornell University, New York, NY, USA) and by selecting for positive cells with 500 µg·mL^−1^ G-418 disulphate (Melford, Ipswich, UK, G0175) in the culture medium.

### 2.2. Generation of P-Rex1-Deficient PC12-S1PR1 Cell Lines

Three sgRNAs were designed using CRISPR sgRNA design software developed by the Feng Zhang laboratory (http://crispr.mit.edu/, accessed on 23 March 2021), for the following criteria: for their ability to direct Cas9 nuclease-mediated double-strand cleavage to exon 4 of the *Prex1* genomic sequence, which encodes for part of the catalytic DH domain; to be positioned directly upstream of a requisite 5′-NGG protospacer adjacent motif (PAM); to have no homologous sites elsewhere in the rat genome. The three sgRNAs were obtained from Sigma Aldrich (Haverhill, UK) and validated in vitro using the Guide-it sgRNA In Vitro Transcription and Screening kit (TakaraBio, Saint-Germain-en-Laye, France, 632636). sgRNA GCCCACGTAGTCGCGCTCGGGTTTT was selected as the most efficacious. PC12-S1PR1 cells were transfected with the sgRNA and with a GeneArt CRISPR Nuclease Vector that expresses orange fluorescent protein (OFP) (ThermoFisher, Loughborough, UK, A21174). Then, 44 h after transfection, cells were resuspended in DMEM supplemented with 1% foetal bovine serum and 1× glutamine, and OFP-positive cells were enriched by fluorescence-activated cell sorting (FACS) in a BD Influx High-Speed Cell Sorter using the 561 nm laser for excitation and 610/20 emission filter to detect OFP. In addition, DAPI DNA dye was used to select viable cells using a 355 nm laser and 530/40 filter, and GFP (from S1PR1-GFP) was detected by 488 nm excitation and 530/30 filter. Single cells were sorted into 96-well plates containing PC12 cell growth medium. Single cells were cultured for clonal expansion in full PC12 cell growth medium supplemented with 500 μg·mL^−1^ G-418 disulphate (Melford, G0175). Clones were screened for targeting success by extracting genomic DNA using tissue lysis buffer (100 mM Tris-HCl, pH 8.5, 200 mM NaCl, 5 mM EDTA, 0.2% SDS, 0.1 mg·mL^−1^ Proteinase K), amplifying the region of interest by PCR with primers ATCCCTGCCCAATACCACAA and GCCCCTTCCCTCATTCTCACT, and sequencing the resulting DNA fragments. Two P-Rex1 deficient cell lines, PC12-S1PR1 P-Rex1 KO1 and KO2, were selected for further analysis and compared to the wild-type PC12-S1PR1 cell line.

To rescue P-Rex1 expression, we stably expressed full-length human P-Rex1 in P-Rex1 deficient PC12-S1PR1 KO1 cells. KO1 cells were seeded into T175 flasks (Nunc, Loughborough, UK, 159910) at a density of 5 × 10^6^ cells per flask. After 24 h, the medium was replaced, and cells were transfected with puromycin-resistant pBABE vector and pcDNA3-mCherry-P-Rex1 using JetPei DNA transfection reagent (Polyplus, 101-10N) according to the manufacturer’s instructions. After 24 h, the medium was replaced with full PC12 medium containing 3 µg·mL^−1^ puromycin dihydrochloride (Sigma, P8833) and 500 µg·mL^−1^ G-418 disulphate (Melford, G0175). After a further 3 days, the surviving cells were collected and passaged for 3 weeks in full PC12 medium containing 1 µg·mL^−1^ puromycin dihydrochloride and 500 µg·mL^−1^ G-418 disulphate. Cells were then resuspended in DMEM supplemented with 1% foetal bovine serum and 1× glutamine, and mCherry-positive cells were enriched by FACS in a BD Influx High-Speed Cell Sorter using the 561 nm laser for excitation and 610/20 emission filter. In addition, DAPI DNA dye was used to select viable cells using a 355 nm laser and 530/40 filter, and GFP (from S1PR1-GFP) was detected by 488 nm excitation and 530/30 filter. mCherry-positive cells were sorted into six-well dishes as four populations depending on the intensity of the mCherry signal. These populations were cultured in full PC12 cell medium and screened for P-Rex1 expression levels by fluorescence microscopy and by Western blotting of total lysates with P-Rex1 antibody. The population with the lowest mCherry signal, which showed P-Rex1 expression levels closest to wild type, was chosen for further analysis.

### 2.3. Western Blotting

Proteins were separated by SDS-PAGE, transferred onto an Immobilon-P PVDF membrane (Millipore), and blotted with the following primary antibodies: P-Rex1 (clone 6F12, kindly provided by Prof. Marcus Thelen, Institute for Research in Biomedicine, Bellinzona, Switzerland, [8]), P-Rex2 (affinity-purified rabbit polyclonal, [29]), S1PR1 (Abcam, Cambridge, UK, ab11424), Rac1 (Millipore, Walford, UK, 05-389), Rac3 (Abcam, ab129062), phospho-Akt S473 (Cell Signaling Technology, London, UK, 9271), Akt (Cell Signaling Technology, 9272), phospho-p38 Mapk (Cell Signaling Technology, 9211), p38 Mapk (Cell Signaling Technology, 9212), phospho-Erk1/2 (Cell Signaling Technology, 9106), Erk1/2 (Cell Signaling Technology, 9102), adenylyl cyclase (AC) 3 (Novus Biologicals, Abingdon, UK, NBP1-92683), AC6 (Abcam, Ab14781); AC9 (Abcam, Ab191423), and β-Cop (M3A5 hybridoma, kindly provided by the late Dr. Thomas Kreis, University of Geneva, Geneva, Switzerland). Secondary antibodies were HRP-conjugated goat anti-mouse IgG (Bio-Rad, Watford, UK, 1706516) or goat anti-rabbit IgG (Bio-Rad, 1706515). Detection was done with Clarity Western ECL Substrate and X-ray film. Where required, membranes were stripped in 25 mM glycine (pH 2.0), 1% SDS and reprobed. For densitometric analysis, films were scanned, and band intensities quantified using ImageJ. To test total protein loading, PVDF membranes were stained with Coomassie blue after blotting.

### 2.4. Rac Activity

Rac activity (GTP-loading) was assayed by Pak-CRIB pulldown using GST-Pak-CRIB immobilised on glutathione-Sepharose 4B, essentially as previously described [1,53]. Briefly, wild-type and P-Rex1-deficient PC12-S1PR1 cells were seeded onto 10 cm culture dishes at 2 × 10^6^ cells per dish, serum-starved overnight in DMEM containing 0.1% fatty acid-free bovine serum albumin, and stimulated with 5 nM sphingosine 1-phosphate (S1P; Sigma S9666) for the indicated periods of time at 37 °C. The medium was aspirated, dishes were placed onto an iced metal tray, and cells were lysed by the addition of 1 mL/dish of ice-cold GST-fish buffer (50 mM Tris-HCl (pH 7.4), 10% glycerol, 100 mM NaCl, 1% NP-40, 2 mM MgCl_2_) supplemented with 2 mM DTT, 0.1 mM PMSF, 10 μg·mL^−1^ leupeptin, 10 μg·mL^−1^ antipain, 10 μg·mL^−1^ aprotinin, 10 μg·mL^−1^ pepstatin-A, with incubation for 5 min on ice. The lysate was transferred into precooled Eppendorf tubes, insoluble material was removed by centrifugation at 12,000× *g* for 5 min at 4 °C, and the supernatant (total lysate) was transferred into fresh precooled tubes. Then, 75 μL aliquots were set aside for total lysate controls. The rest of the sample was incubated with 10 μL PAK-CRIB-GST beads for 15 min on ice with end-over-end rotation. Beads were washed thrice in ice-cold GST-fish buffer. Proteins were denatured in boiling SDS sample buffer, resolved by SDS-PAGE, and Western blotted for GTP-bound active and total Rac1 and Rac3. GTP-Rac and total Rac blots were processed in parallel on the same films to allow direct comparison of the level of active Rac in the cell.

To test the specificity of the Rac3 antibody used in the Rac3 activity assays, wild-type PC12-S1PR1 cells were seeded into six-well dishes (Nunc, 140675) at 3 × 10^5^ cells per well, grown for 24 h, and then cultured in antibiotic-free medium for a further 6 h. Cells were transfected with 5 or 25 nM ON-TARGETplus Rat Rac3 siRNA (688319) or ON-TARGETplus Non-targeting Control Pool (Horizon, L-114657-00-0005 and D-001810-10-05, respectively) using DharmaFECT 1 Transfection Reagent (Horizon, Cambridge, UK, T-2001-0), according to the manufacturer’s instructions, or were mock-treated. After 48 h, cells were lysed and analysed for the expression levels of endogenous Rac1 and Rac3 by Western blotting.

### 2.5. Akt, p38 Mapk, and Erk Activity

To determine the activity of Akt, p38 Mapk, and Erk, the same total lysates that were prepared for Rac activity assays as described above were blotted with antibodies against the phosphorylated active forms and the total levels of these proteins. Activity was quantified by dividing the phospho-signal from ImageJ analysis by the total signal for each protein.

### 2.6. cAMP Production

Wild-type and P-Rex1-deficient PC12-S1PR1 cells were seeded into six-well dishes (Nunc, 140675) at 3 × 10^5^ cells per well, grown for 24 h, then serum-starved overnight in DMEM containing 0.1% fatty acid-free bovine serum albumin, and stimulated with 5 nM S1P and/or 1 µM forskolin (Bio-Techne, Abingdon, UK, 1099/10) or were mock-stimulated, for 10 min at 37 °C. The medium was aspirated, and 200 µL of 0.1% HCl, 0.1% Triton X-100 was added to lyse the cells for 10 min at RT. The supernatant was collected by centrifugation at 700× *g* for 5 min. cAMP levels were measured using the Direct cAMP ELISA kit (Enzo, Exeter, UK, ADI-900-066) according to the manufacturer’s instructions. Total protein levels were measured using DC Protein Assay (Bio-Rad, 500-0116) according to the manufacturer’s instructions. In some experiments, cells were treated with the phosphodiesterase inhibitor IBMX prior to cell lysis and cAMP assays, by adding 100 µM IBMX (Enzo Life Sciences, BML-PD140-0200) to the starvation medium for 1 h before stimulating the cells and during stimulation.

### 2.7. Cell Morphology

Wild type and P-Rex1 deficient PC12-S1PR1 cells were seeded onto glass coverslips, grown for 24 h, then serum-starved overnight in DMEM containing 0.1% fatty acid-free bovine serum albumin, stimulated with S1P at the indicated concentrations for 10 min, and fixed with 4% paraformaldehyde, 50 mM Pipes, pH 6.5, 1 mM EGTA, 10 mM MgCl_2_ for 15 min at RT. Cells were washed thrice in phosphate-buffered saline (PBS), stained with Hoechst 33342 DNA dye, and mounted onto slides using Aqua-Poly/Mount (Polysciences, Hirschberg, Germany, 18606-20). Cells were imaged using a Nikon AR1 confocal microscope (60× objective). Image analysis was done using CellProfiler software (Broad Institute, Cambridge, MA, USA) by making masks for each cell to determine the cell perimeter and cell area.

### 2.8. Cell Growth and Viability

Wild-type and P-Rex1-deficient PC12-S1PR1 cells were seeded into 12-well plates (Nunc, 150628) in full growth medium at a density of 2 × 10^4^ cells per well. The medium was changed on day 3. Once every 24 h, for 6 days, aliquots of cells were tested by trypan blue exclusion assay using 0.4% trypan blue dye and cell counting by haemocytometer, to assess growth and viability.

### 2.9. Cell Cycle

Wild-type PC12-S1PR1 cells, P-Rex1-deficient PC12-S1PR1 cells, or P-Rex1-deficient PC12-S1PR1 cells reconstituted with human P-Rex1 were seeded into T175 flasks (Nunc, 159910) at a density of 5 × 10^6^ per flask. After 20 h, the medium was collected, and cells were dislodged using TrypLE Express (Thermo Fisher, 12604013) and pooled with the collected medium. Cells were sedimented at 433× *g* for 5 min at RT and washed once in PBS. Cells were resuspended in ice-cold 70% ethanol/PBS and incubated on ice for 30 min for fixing and permeabilisation, washed in PBS, and stained for 30 min at 37 °C using 5 μg/mL DAPI DNA dye (Sigma D9542) in PBS containing 100 μg·mL^−1^ RNase (Roche, Welwyn Garden City, UK, 109142001). Samples were filtered through 50 µM cell strainers (CellTrics, Milton Keynes, UK, 04-004-2327) prior to analysis by flow cytometry using a FACSCalibur analyser. The intensity of DAPI staining was measured for 10^4^ cells per sample and used to determine the cell-cycle phase for each cell using FlowJo software version 10 (FloJo LLC, Ashland, OR, USA).

### 2.10. Experimental Design and Statistical Analysis

Data were tested for normality of distribution to determine if parametric or nonparametric methods of analysis were appropriate. For comparison between two groups, unpaired Student’s *t*-test was used, whereas, for comparison among multiple groups or for longitudinally observed parameters between different groups, two-way ANOVA was used with repeated measures followed by post hoc test with Sidak’s multiple comparisons correction. Parameters with values of *p* ≤ 0.05 were considered to differ significantly. In the figures, *p*-values are specified; alternatively, * indicates *p* ≤ 0.05, ** indicates *p* ≤ 0.01, *** indicates *p* ≤ 0.001, and **** indicates *p* ≤ 0.0001 for comparisons between wild-type and P-Rex1-deficient groups; ^#^ indicates *p* ≤ 0.05, ^##^ indicates *p* ≤ 0.01, and ^###^ indicates *p* ≤ 0.001 for the evaluation of concentration-dependent changes within the same genotype. Where relevant, data that did not show significant differences between wild-type and P-Rex1-deficient groups are highlighted as ‘ns’. Results are presented as the mean ± standard error of the mean (SEM). The number of experimental repeats is indicated in the figure legends. Statistical analysis and plotting of graphs were performed in GraphPad Prism version 8.3.1 (GraphPad Software, San Diego, CA, USA).

## 3. Results

### 3.1. Generation of P-Rex1-Deficient PC12-S1PR1 Cell Lines

**Figure 1:**
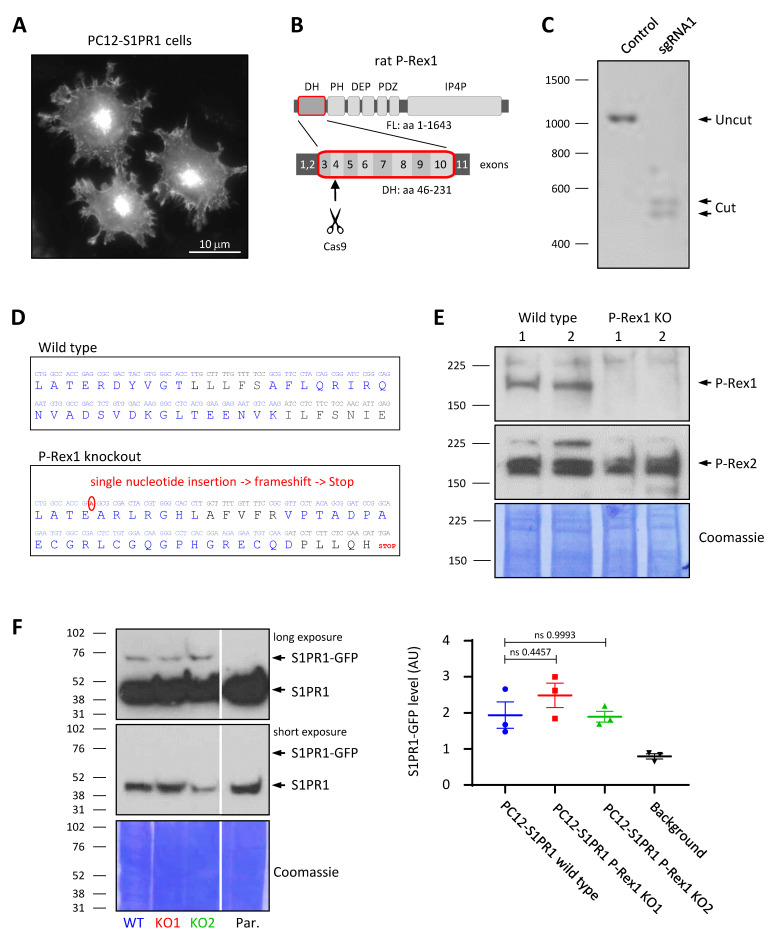
Generation of P-Rex1-deficient PC12-S1PR1 cells. (**A**) Representative wide-field fluorescence image of PC12-S1PR1 cells. Shown is the GFP signal from stably expressed S1PR1-GFP. (**B**) P-Rex1 domain structure, exon structure of the catalytic DH domain in rat, highlighting exon 4 which we targeted to generate P-Rex1 deficiency in PC12-S1PR1 cells by CRISPR/Cas9-mediated gene editing. (**C**) In vitro quality testing of the sgRNA selected for CRISPR/Cas9-mediated targeting of P-Rex1. The DNA gel shows efficient cutting of the target DNA by Cas9 in the presence of the sgRNA. (**D**) cDNA and amino-acid sequence showing the region of interest from exon 4 to the start of exon 7 of rat P-Rex1, from wild-type and P-Rex1-deficient PC12-S1PR1 cells. Blue and black letters mark different exons. CRISPR/Cas9 gene editing inserted a single nucleotide into exon 4 of P-Rex1, causing frameshift and premature Stop. (**E**) Western blot analysis of wild-type and P-Rex1-deficient PC12-S1PR1 cell lines for P-Rex1 and P-Rex2 expression; Coomassie staining of the membrane was used to control for protein loading. (**F**) P-Rex1 deficiency did not affect S1PR1 levels in PC12-S1PR1 cell lines. ‘WT’ denotes wild-type PC12-S1PR1 cells. ‘KO1’ and ‘KO2’ denote successfully targeted P-Pex1-deficient PC12-S1PR1 cell lines. ‘Par’ denotes parental PC12 cells without S1PR1-GFP. The left-hand panel shows representative S1PR1 Western blots and the Coomassie-stained membrane. The white line denotes cropping of irrelevant lanes from the membrane. The right-hand panel shows quantification by densitometric analysis of blots from three independent experiments. Data are presented as the mean ± SEM; each symbol represents one experiment. Statistical analysis involved one-way ANOVA with Sidak’s multiple comparisons test; ‘ns’ denotes data which were not significantly different.

To study the role of P-Rex1 in GPCR-mediated neuronal signalling and responses, we first generated a PC12 cell line that stably expresses a C-terminally GFP-tagged form of the GPCR S1PR1 (Figure 1A). We then deleted P-Rex1 from these PC12-S1PR1 cells by CRISPR/Cas9 knockout. For this, sgRNAs were designed to target exon 4 of P-Rex1, which codes for part of the catalytic DH domain (Figure 1B), and were tested in vitro for efficacy. The best sgRNA (Figure 1C) was selected to guide Cas9 nuclease to induce double-strand breaks in the target sequence. Clonal cell lines were generated, and P-Rex1 deficiency was confirmed by both DNA sequencing and Western blotting (Figure 1D,E). DNA sequencing confirmed that the gene editing had introduced a single nucleotide into exon 4, causing a frameshift, premature Stop, and P-Rex1 knockout (Figure 1D). P-Rex2 expression was not affected by the P-Rex1 deficiency (Figure 1E). Two P-Rex1-deficient PC12-S1RP1 cell lines, PC12-S1PR1 P-Rex1 KO1 and KO2, were selected for analysis and compared to wild-type PC12-S1PR1 cells. To test if P-Rex1 deficiency affected the expression level of S1PR1, total cell lysates from these cell lines and from parental PC12 cells were compared by Western blotting. This showed that the expression level of S1PR1-GFP in all PC12-S1PR1 cell lines was negligible compared to that of endogenous S1PR1 in parental PC12 cells, and P-Rex1 deficiency did not affect the expression of S1PR1-GFP (Figure 1F).

### 3.2. P-Rex1 Deficiency Reduces the S1P-Stimulated Activation of Rac1 and Basal Rac3 Activity in PC12-S1PR1 Cells

**Figure 2:**
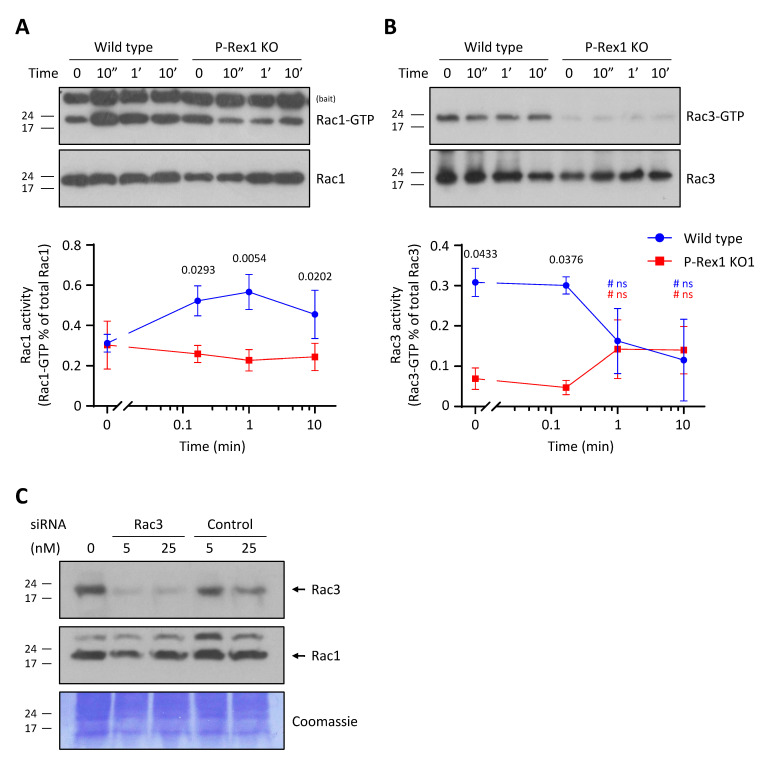
P-Rex1 deficiency reduces the S1P-stimulated activation of Rac1 and basal Rac3 activity in PC12-S1PR1 cells. (**A**) P-Rex1 is required for the S1P-stimulated activation of Rac1 in PC12-S1PR1 cells. Wild-type and P-Rex1-deficient PC12-S1PR1 cells were serum-starved overnight and stimulated with 5 nM S1P for the indicated periods of time at 37 °C prior to cell lysis and isolation of active, GTP-bound Rac by Pak-CRIB pulldown assay. Top panels are representative Western blots showing the level of active, GTP-loaded Rac1 compared to total Rac1 in 1% of the cell lysate, processed on the same film. Bottom panel shows the quantification by densitometric analysis. Data are from six independent experiments and are presented as the mean ± SEM. Statistical analysis involved two-way ANOVA with Sidak’s multiple comparisons test. (**B**) P-Rex1 is required for basal Rac3 activity in PC12-S1PR1 cells. The same cell lysates as in (**A**) were analysed for Rac3 activity. Top panels are representative Western blots showing active, GTP-loaded Rac3 from Pak-CRIB pulldown assay compared to total Rac3 level in 1% of the cell lysate, processed on the same film. Bottom panels show quantification by densitometric analysis. Data are from three independent experiments and are presented as the mean ± SEM. Statistical analysis involved two-way ANOVA with Sidak’s multiple comparisons test; ‘# ns’ denotes no significant differences between S1P-stimulated and control conditions within each genotype. (**C**) Rac3 antibody specificity. Wild-type PC12-S1PR1 cells were treated with the indicated doses of Rac3 siRNA or control siRNA, and total cell lysates were Western blotted for endogenous Rac1 and Rac3; Coomassie staining of the membrane was used as a loading control.

PC12 cells express both the ubiquitous Rac1 isoform of Rac GTPases and the neuronal Rac3 isoform, and these isoforms are known to have opposite roles in regulating neuronal cytoskeletal dynamics [37]. Previous studies have reported that P-Rex1 can act as a Rac1-GEF [28] or a Rac3-GEF [36] in PC12 cells stimulated with the growth factor NGF, although the ability to activate Rac3 has to date only been tested for exogenously expressed GTPase. In order to assess the substrate specificity of P-Rex1 in the context of S1P stimulation, we measured the activities (GTP loading) of endogenous Rac1 and Rac3 in S1P-stimulated wild-type and P-Rex1-deficient PC12-S1PR1 cells by Pak-CRIB pulldown assay. S1P stimulation rapidly induced the activation of endogenous Rac1 in wild-type PC12-S1PR1 cells, with a peak at 1 min after stimulation. In contrast, no activation of Rac1 was observed in P-Rex1-deficient PC12-S1PR1 cells (Figure 2A). These data demonstrate that P-Rex1 acts as a Rac1-GEF in neuronal GPCR signalling, as well as in growth factor signalling, as previously reported [28]. As S1P-stimulated Rac1 activity was abolished in P-Rex1-deficient cells, it appears that P-Rex1 is the major Rac1-GEF in this GPCR signalling pathway.

Like Rac1, only a small proportion of endogenous Rac3 (around 0.3%) was constitutively GTP-loaded in serum-starved and mock-treated wild-type PC12-S1PR1 cells. However, unlike for Rac1, S1P stimulation did not increase Rac3 activity. Rather, it appeared to inhibit Rac3 at later timepoints, but this inhibition was not observed consistently, meaning that Rac3 activity overall remained unaffected by S1P (Figure 2B). The difference between Rac1 and Rac3 activity confirms previous reports that Rac1 and Rac3 signal through distinct mechanisms, in accordance with their diverging neuronal functions [37]. Importantly, P-Rex1-deficient PC12-S1PR1 cells had significantly lower levels of basal Rac3 activity than wild-type PC12-S1PR1 cells, as well as lower Rac3 activity at early timepoints of S1P stimulation. To verify the specificity of the Rac3 antibody used to measure Rac3 activity, we depleted endogenous Rac3 in wild-type PC12-S1PR1 cells using siRNA. This confirmed that the Rac3 antibody recognises Rac3 but not Rac1 (Figure 2C), validating the Rac3 activity assays.

Together, these results demonstrate that P-Rex1 is both a Rac1-GEF and a Rac3-GEF in neuronal PC12 cells. Its Rac1-GEF activity is required during S1P stimulation, whereas it Rac3-GEF activity confers low-level basal GTP-loading of Rac3, independently of S1P.

### 3.3. P-Rex1 Deficiency Increases Akt Activity upon S1P Stimulation without Affecting P38 Mapk or Erk Activities in PC12-S1PR1 Cells

**Figure 3:**
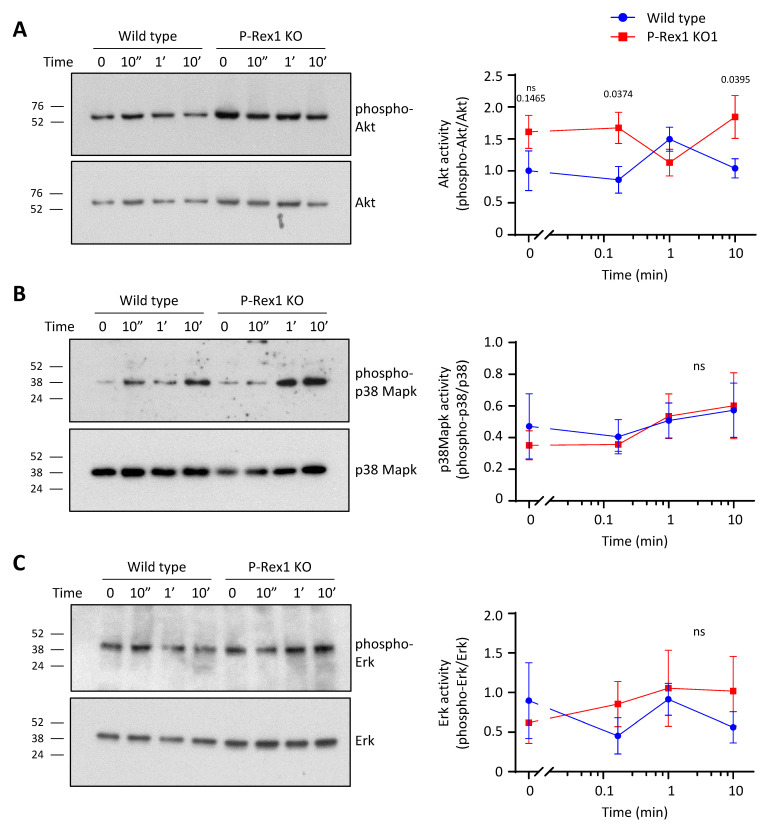
P-Rex1 deficiency increases Akt activity upon S1P stimulation without affecting p38 Mapk or Erk activities in PC12-S1PR1 cells. The same lysates of wild-type and P-Rex1-deficient PC12-S1PR1 cells as shown in Figure 2 were analysed for the activities of Akt, p38 Mapk, and Erk. (**A**) P-Rex1 deficiency increases Akt activity upon S1P stimulation of PC12-S1PR1 cells. Representative Western blots and quantification of Akt activity as phospho-Akt-S473 signal divided by total Akt signal are shown. (**B**,**C**) P-Rex1 deficiency does not affect p38 Mapk activity (**B**) or Erk activity (**C**) in S1P-stimulated PC12-S1PR1 cells. Representative Western blots and quantification of p38 Mapk activity, expressed as phospho-p38 signal divided by total p38 Mapk signal (**B**), and of Erk activity, expressed as phospho-Erk signal divided by total Erk signal (**C**), are shown. Data in (**A**–**C**) are from five independent experiments and are presented as the mean ± SEM. Statistical analysis involved two-way ANOVA with Sidak’s multiple comparisons test. ‘ns’ denotes data that were not significantly different between genotypes.

To test whether P-Rex1 deficiency affected other major signalling pathways under the same conditions that revealed its role in the regulation of Rac1 and Rac3, we tested total lysates from the Rac activity assays for the activities of Akt, p38 Mapk, and Erk by phospho-Western blotting. None of these pathways were significantly activated by S1P stimulation in wild-type PC12-S1PR1 cells under the same conditions that produced robust activation of Rac1 (Figure 3). However, P-Rex1-deficient PC12-S1PR1 cells showed increased Akt activity (phosphorylation of S473) upon S1P stimulation (Figure 3A). Mock-stimulated P-Rex1 deficient cells also showed higher Akt activity than wild-type in five out of six experiments, but this did not reach statistical significance. Akt expression levels were not affected. Together, these data suggest that endogenous levels of P-Rex1 serve to limit Akt activity during GPCR signalling. In contrast to Akt, the activities of p38 Mapk and Erk were not affected by P-Rex1 deficiency under the conditions tested (Figure 3B,C).

### 3.4. P-Rex1 Deficiency Constitutively Increases cAMP Levels in PC12-S1PR1 Cells, without Increased Expression of Major Neuronal Adenylyl Cyclases

**Figure 4:**
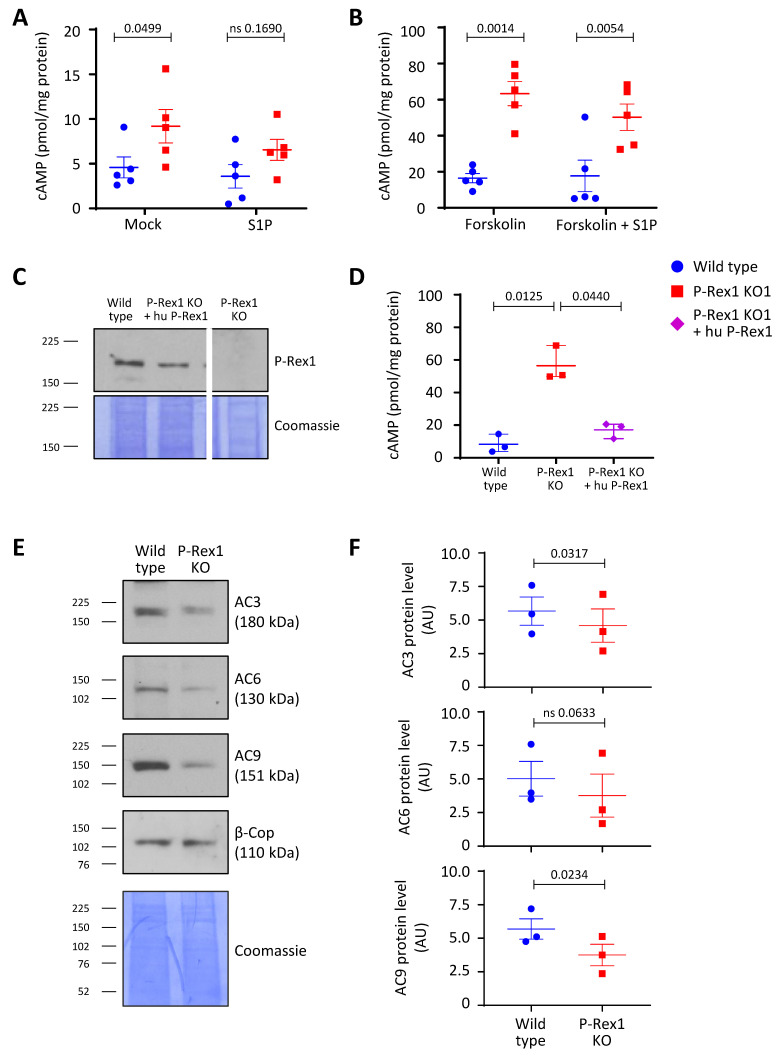
P-Rex1 deficiency constitutively increases cAMP levels in PC12-S1PR1 cells, while decreasing the levels of major neuronal adenylyl cyclases. (**A**,**B**) P-Rex1 deficiency constitutively increases cAMP levels in PC12-S1PR1 cells. (**A**) Wild-type and P-Rex1-deficient PC12-S1PR1 cells were serum-starved overnight, stimulated with 5 nM S1P for 10 min or mock-stimulated, and lysed, while cAMP levels in the cell lysate were analysed by ELISA. cAMP levels were normalised to total protein. (**B**) Cells were treated as in (**A**) except that 1 µM forskolin was added during the 10 min S1P or mock stimulation. cAMP levels were determined as in (**A**). Data in (**A**,**B**) are from five independent experiments and are presented as the mean ± SEM; each symbol is the mean of one experiment. Statistical analysis involved two-way ANOVA with Sidak’s multiple comparisons tests; ‘ns’ denotes nonsignificant differences. (**C**) P-Rex1 rescued cells: P-Rex1 Western blot of total lysates from wild-type and P-Rex1-deficient PC12-S1PR1 cells, as well as from P-Rex1 deficient PC12-S1PR1 cells reconstituted with stably expressed human full-length P-Rex1 (hu P-Rex1). The white line denotes cropping of irrelevant lanes from the membrane. Coomassie staining of the membrane was used as a loading control. (**D**) cAMP production was tested in wild-type and P-Rex1-deficient PC12-S1PR1 cells, as well as in P-Rex1-deficient PC12-S1PR1 cells reconstituted with human full-length P-Rex1 as in (**C**), in the presence of forskolin as in (**B**). Data are from three independent experiments and are presented as the mean ± SEM; each symbol is the mean of one experiment. (**E**,**F**) P-Rex1 deficiency decreases the protein level of adenylyl cyclases. (**E**) Representative Western blots of whole-cell lysates of wild-type and P-Rex1-deficient PC12-S1PR1 cells for neuronal adenylyl cyclases AC3, AC6 and AC9. β-Cop and Coomassie staining were used as loading controls. (**F**) Quantification of western blots as in (**E**). Data in (**F**) are from three independent experiments and are presented as the mean ± SEM; each symbol is one experiment. Statistical analysis involved paired Student’s *t*-test; ‘ns’ denotes nonsignificant differences.

A complex relationship has previously been reported between the P-Rex1 and PKA signalling pathways. However, the possibility that P-Rex1 may regulate the upstream signal of PKA signalling, i.e., the cellular level of cAMP, has never been investigated. We found that P-Rex1 deficiency increased cAMP levels under both basal and S1P-stimulated conditions in PC12-S1PR1 cells (Figure 4A). As cAMP is the major direct activator of PKA, this suggests that P-Rex1 may regulate PKA signalling by controlling its upstream signal, as well as by regulating the subcellular localisation of PKA, as previously reported [46].

Given that S1PR1 is a Gαi-coupled GPCR, S1P stimulation leads to the inhibition of adenylyl cyclases (the enzymes that produce cAMP) and, hence, to a reduction in cellular cAMP levels. Indeed, cAMP levels tended to be lower upon S1P stimulation of PC12-S1PR1 cells, but they remained elevated in P-Rex1-deficient cells compared to wild-type. Adenylyl cyclases can be directly activated by treatment with forskolin. We treated PC12-S1PR1 cells with forskolin to see if this would overcome the effect of P-Rex1 deficiency. Forskolin treatment increased cellular cAMP levels in wild-type PC12-S1PR1 cells, as expected. However, cAMP levels increased accordingly in P-Rex1-deficient PC12-S1PR1 cells (Figure 4B). Essentially the same results were obtained with our second P-Rex1-deficient PC12-S1PR1 cell line, P-Rex1 KO2 (data not shown). These results suggested that P-Rex1 deficiency either increased the production of cAMP, further than was achieved by forskolin treatment, or decreased the metabolism of cAMP.

To ascertain that the altered cAMP levels were not an off-target effect of the CRISPR P-Rex1 knockout, we stably expressed full-length human P-Rex1 in the P-Rex1-deficient PC12-S1PR1 cells to a similar level as endogenous P-Rex1 in wild-type PC12-S1PR1 cells (Figure 4C). Under these conditions, cAMP levels were rescued to those seen in wild-type cells, confirming that control of cAMP levels is a bona fide function of P-Rex1 (Figure 4D).

We tested whether the increased cAMP levels derived from elevated expression levels of adenylyl cyclase in P-Rex1-deficient PC12-S1PR1 cells. Mammals have 10 different isoforms of adenylyl cyclases, and the major isoforms expressed in neuronal PC12 cells are adenylyl cyclases 3, 6, and 9 [54]. Against our expectations, Western blotting of total cell lysates showed that P-Rex1 deficiency decreased the level of adenylyl cyclase isoforms AC3 and AC9 compared to wild-type cells, and it also had a tendency to decrease the level of AC6 (Figure 4E,F). Decreased expression of these adenylyl cyclases was also seen in the second P-Rex1-deficient PC12-S1PR1 cell line, KO2 (data not shown). Hence, P-Rex1 deficiency does not lead to increased protein levels of adenylyl cyclases. We tested next whether the metabolism of cAMP is affected, by treating cells with IBMX, a potent inhibitor of the phosphodiesterase enzymes that metabolise cAMP to AMP. We thought that P-Rex1 deficiency may somehow lower the activity of phosphodiesterases, and that the KO cells might, therefore, be less sensitive to IBMX than wild-type cells. However, this did not appear to be the case (data not shown). Overall, it seems therefore more likely that P-Rex1 limits the catalytic activity of neuronal adenylyl cyclases, and that P-Rex1-deficient cells downregulate the expression of adenylyl cyclases in an attempt to counteract the elevated cAMP production.

### 3.5. P-Rex1 Is Required for the Maintenance of Neurite-like Protrusions and Spreading in S1P-Stimulated PC12-S1PR1 Cells

**Figure 5:**
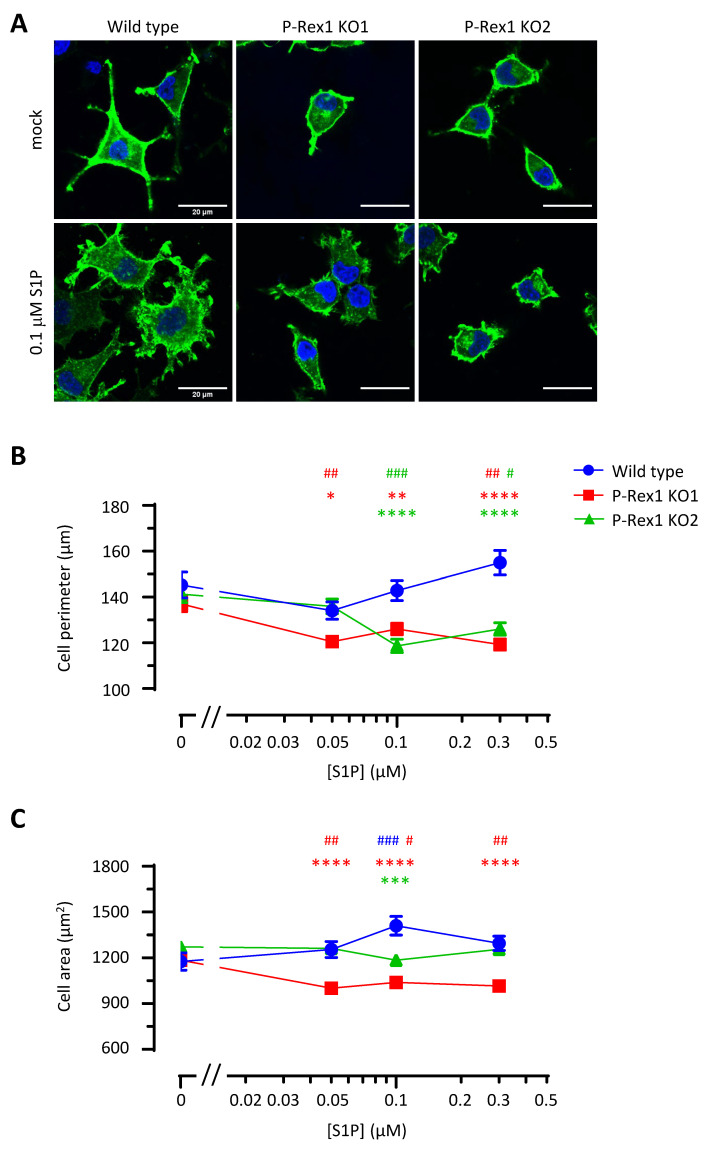
P-Rex1 is required for the maintenance of neurite protrusions and spreading in S1P-stimulated PC12-S1PR1 cells. (**A**) Representative confocal images of wild-type and P-Rex1-deficient PC12-S1PR1 cells grown on glass coverslips for 24 h, serum-starved overnight, and stimulated with indicated concentrations of S1P or mock stimulated, for 10 min prior to fixation. The green signal is GFP from S1PR1-GFP, while the blue signal is from Hoechst staining of the nucleus. Scale bar = 20 µm. (**B**,**C**) Quantification of cell perimeter (**B**) and cell area (**C**) of cells treated as in (**A**). For image analysis, cell masks were applied using CellProfiler software, and cell perimeter and area were calculated from the masks. Data are presented as the mean ± SEM of cells pooled from three independent experiments. At least 250 cells were analysed per condition. Outliers were identified using ROUT in GraphPad Prism 8.3.1 and were removed prior to statistical analysis. Statistical analysis involved two-way ANOVA with Dunnett’s multiple comparisons test. Asterisks denote differences between P-Rex1-deficient and wild-type cells, whereas hashes denote S1P-dependent differences within one genotype.

P-Rex1 is known to regulate PC12 cell morphology in response to NGF stimulation. However, the exact function of P-Rex1 in this process remains to be elucidated, as P-Rex1 was shown to either stimulate the formation of neurite-like protrusions during acute NGF stimulation or cause neurites to retract during prolonged NGF treatment [28,36]. A role for P-Rex1 in GPCR-mediated effects on PC12 cell morphology has not previously been investigated. We assessed the morphology of PC12-S1PR1 cells upon S1P stimulation by confocal fluorescence imaging (Figure 5A) and quantified the cell perimeter and cell area by image analysis (Figure 5B). Upon plating on glass, PC12-S1PR1 cells formed neurite-like protrusions, which seemed to increase but were not significantly affected by S1P stimulation in wild-type PC12-S1PR1 cells. However, P-Rex1 deficiency caused a progressive loss of these neurite-like protrusions with increasing concentrations of S1P stimulation, as evidenced by a reduction in cell perimeter (Figure 5B). This was seen in both P-Rex1-deficient cell lines. Hence, P-Rex1 serves to maintain PC12 cell protrusions during S1P signalling. Furthermore, S1P stimulation increased cell spreading in wild-type PC12-S1PR1, as evidenced by the increase in cell area. This S1P-induced cell spreading was abolished by the P-Rex1 deficiency, albeit more so in one than the other P-Rex1 KO cell line (Figure 5C). Together, P-Rex1 is required for the maintenance of neurite-like protrusions and for the S1P-dependent spreading of neuronal cells.

### 3.6. P-Rex1 Deficiency Reduces the Proliferation and Cell-Cycle Progression of PC12-S1PR1 Cells

**Figure 6:**
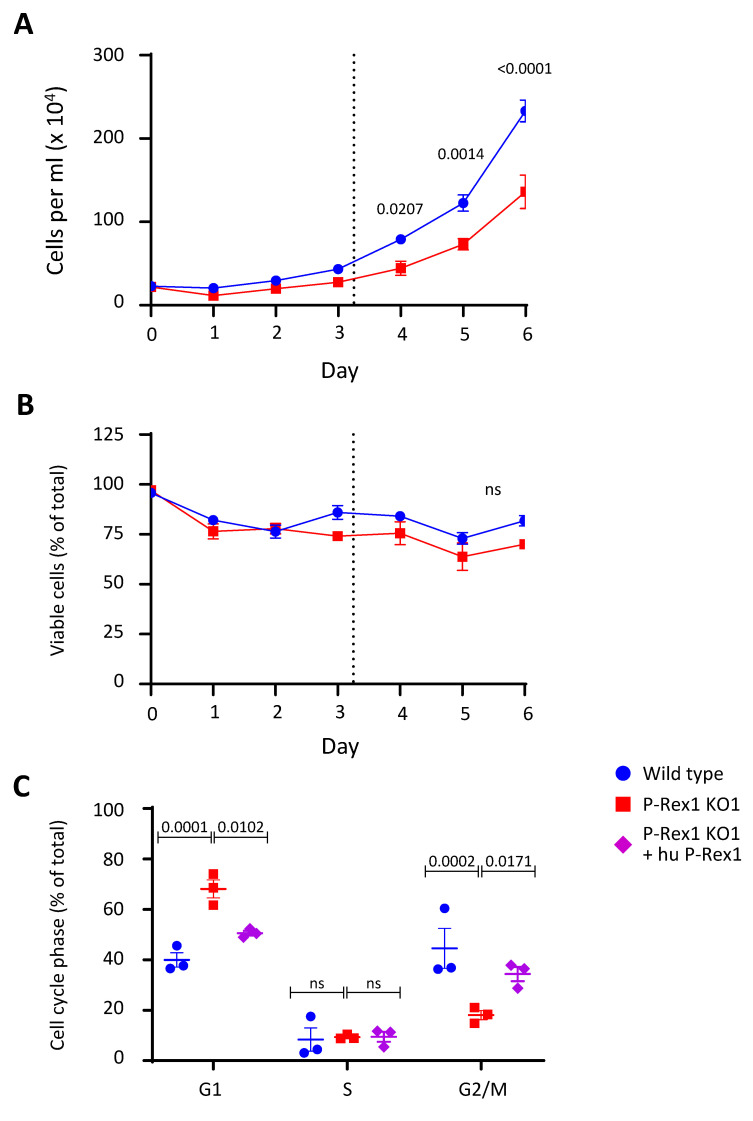
P-Rex1 deficiency reduces PC12-S1PR1 cell proliferation and cell-cycle progression. (**A**,**B**) P-Rex1 deficiency reduces the growth rate of PC12-S1PR1 cells without affecting cell viability. Wild-type and P-Rex1-deficient PC12-S1PR1 cells were grown in full medium. Aliquots of cells were counted by haemocytometer to assess growth (**A**) or tested by trypan blue exclusion assay for viability (**B**) at the indicated timepoints. Data in (**A**,**B**) are the mean ± SEM of three independent experiments. Statistical analysis involved two-way ANOVA with Sidak’s multiple comparisons tests. (**C**) P-Rex1 deficiency caused an accumulation of cells in G1 cell-cycle phase. Wild-type and P-Rex1-deficient PC12-S1PR1 cells, as well as P-Rex1-deficient PC12-S1PR1 cells reconstituted with human full-length P-Rex1 (hu P-Rex1), were grown in full medium, fixed and permeabilised in 70% ethanol, and stained with DAPI to determine stages of the cell cycle and levels of apoptosis by flow cytometry. Data are from three independent experiments and are presented as the mean ± SEM; each symbol is one experiment. Statistical analysis involved two-way ANOVA with Sidak’s multiple comparisons test; ‘ns’ denotes nonsignificant differences.

P-Rex1 is known to control proliferation and cell-cycle progression in a number of different cell types, mainly cancer cells [2,3]. However, the role of P-Rex1 in neuronal proliferation and cell-cycle progression has not yet been investigated. We assessed the growth rates of wild-type and P-Rex1-deficient PC12-S1PR1 cells, which revealed that P-Rex1 deficiency caused slower growth (Figure 6A). This was seen despite normal viability of P-Rex1-deficient PC12-S1PR1 cells (Figure 6B). Essentially the same was seen in our second P-Rex1-deficient PC12-S1PR1 cell line, KO2 (data not shown). These data suggested that P-Rex1 is required for the proliferation of neurons. We evaluated cell-cycle progression and apoptosis using DAPI staining and flow cytometry. Sub-G1 cells were negligible in both wild-type and P-Rex1-deficient PC12-S1PR1 cells, suggesting that P-Rex deficiency did not induce apoptosis, in accordance with the normal viability.

However, P-Rex1 deficiency did induce a marked increase in the proportion of cells in G1 and a concomitant decrease in G2/M phase (Figure 6C). Hence, P-Rex1 deficiency causes an impairment in cell-cycle progression in PC12-S1PR1 cells, which is likely to be the underlying mechanism of the reduced proliferation.

## 4. Discussion

Our study identified new functional roles of P-Rex1 in neuronal signalling and responses. We showed that P-Rex1 is required for the S1P-stimulated activation of Rac1 and Akt, for basal Rac3 activity, and for constitutive cAMP production in neuronal PC12-S1PR1 cells. We also showed that P-Rex1 is required for the maintenance of neurite protrusions and for cell spreading upon S1P stimulation, as well as for the cell-cycle progression and proliferation of PC12-S1PR1 cells.

We deleted P-Rex1 from PC12-S1PR1 cells in order to provide a model for studying the role of endogenous P-Rex1 in neuronal sphingosine 1-phosphate receptor signalling. We showed that P-Rex1 can act both as a Rac1 and a Rac3 GEF in these cells, resolving previous controversy in the literature suggesting that P-Rex1 would activate one or the other of these Rac GTPases upon PC12 stimulation with NGF [28,36]. We show that P-Rex1 is required for the S1P-stimulated activation of Rac1 and for basal Rac3 activity. As P-Rex1 deficiency abolished the S1P-stimulated activation of Rac1, we suggest that P-Rex1 is the major Rac-GEF in this pathway. Our study is the first to demonstrate that P-Rex1 acts as a GEF for endogenous Rac3. S1P stimulation increased Rac1 but not Rac3 activity in wild type cells; in fact, it appeared to reduce Rac3 GTP-loading in some experiments. These data support previous reports which demonstrated opposing functional roles of neuronal Rac1 and Rac3 [37] and show additionally that neuronal Rac1 and Rac3 also respond in an opposing manner to upstream GPCR signals, although both isoforms are subject to regulation by P-Rex1. The mechanisms underlying the different cellular roles of Rac1 and Rac3 are incompletely understood, but they appear to be connected to sequence variations in the C-termini of the GTPases dictating differential subcellular localisations and opposing effects on integrin-dependent responses [37]. Global Rac1 deficiency in mice is embryonic lethal, and targeted deletions of Rac1 in the nervous system have demonstrated that Rac1 is essential for brain development. For example, deletion of Rac1 in the forebrain results in loss of neural progenitor cells, associated with increased cell-cycle progression and apoptosis, leading to microcephaly [55]. Interestingly, missense mutations in Rac1 were recently identified to be associated with several human developmental disorders, including microcephaly, through dominant-negative effects of the Rac1 mutants on neuronal development [56]. In contrast to Rac1, Rac3 is nonessential for development, and Rac3-deficient mice show relatively mild behavioural abnormalities of superior motor coordination and motor learning [57]. Direct comparison of mice with postmitotic neuronal Rac1 deficiency and with Rac3 deficiency has identified a number of both diverging and synergistic roles for these GTPases in the development of cortical and hippocampal interneurons [58].

Our investigation of the effects of P-Rex1 on the morphology of PC12-S1PR1 cells showed that P-Rex1 deficiency caused a loss of neurite-like protrusions and cell spreading upon S1P stimulation, suggesting that endogenous P-Rex1 serves to maintain neurites. Previously P-Rex1 was shown to either stimulate the formation of neurite-like protrusions during acute NGF stimulation, dependent on the Rac1-GEF activity of P-Rex1, or cause neurites to retract during prolonged NGF treatment, linked to Rac3-GEF activity [28,36]. In general, Rac1 activity promotes protrusion formation, whereas Rac3 activity favours cytoskeletal retractions and rounding in neuronal cells [37]. As P-Rex1 deficiency caused loss of protrusions in S1P-stimulated PC12-S1PR1 cells, it seems that its Rac1-GEF activity predominates over its Rac3-GEF activity in this context, consistent with the loss of Rac3 activity seen upon S1P stimulation of wild-type PC12-S1PR1 cells.

We examined several other pathways in addition to Rac, under the same conditions that induced the S1P-stimulated activation of Rac1. In wild-type PC12-S1PR1 cells, the activities of Akt, p38 Mapk, and Erk were unaffected under these conditions. However, P-Rex1-deficient PC12-S1PR1 cells showed significantly increased Akt activity upon S1P stimulation, which suggested that endogenous P-Rex1 levels limit Akt activation during GPCR signalling. This may be caused by the known direct interaction of P-Rex1 with the kinase mTOR, which makes P-Rex1 a component of the mTORC2 complex. mTORC2 functions as the PIP_3_-dependent kinase 2 that phosphorylates Akt on serine 473 to confer full activation of Akt pathway activity. Our data, therefore, suggest that endogenous P-Rex1 limits mTORC2 activity [59]. It would be interesting to investigate this possibility with in vitro assays to test the effects of P-Rex1 on mTOR kinase activity in the future.

PKA is an important negative regulator of P-Rex1 Rac-GEF activity; inversely, P-Rex1 can regulate the subcellular localisation of PKA [39,46,47]. We investigated here the possibility that P-Rex1 may also affect the upstream activating signal of PKA, i.e., cAMP production. Indeed, P-Rex1 deficiency caused a constitutive increase in cAMP levels in PC12-S1PR1 cells. This was seen despite major neuronal adenylyl cyclases being downregulated, if at all, and it persisted upon S1P stimulation and upon forskolin-induced direct stimulation of adenylyl cyclase activity. Furthermore, inhibition of phosphodiesterases, the enzymes that metabolise cAMP, did not abrogate the increased cAMP levels in P-Rex1-deficient cells. We considered whether P-Rex1 deficiency might affect the sequestration of cAMP, for example, cAMP binding to PKA; however, as we measured total cellular cAMP levels, this possibility seems unlikely. Therefore, we propose that endogenous P-Rex1 might serve to limit adenylyl cyclase activity, with P-Rex1 deficiency causing supra-activation of these enzymes. Future investigations will be required to elucidate the mechanisms underlying P-Rex1-dependent cAMP production and the consequences for PKA signalling and cell responses.

The P-Rex1-dependent regulation of cell-cycle progression and/or cell proliferation has previously been reported, mostly in cancer cells but also in melanoblasts and endothelial cells, and it is often found associated with altered Erk activity and expression levels of cell-cycle regulators [16,60,61,62]. However, P-Rex1 does not seem to affect these processes universally, as others have reported a lack of effect of P-Rex1 on the proliferation of breast cancer cells [63]. We found that P-Rex1 deficiency reduced the growth of PC12-S1PR1 cells, without causing apoptosis or affecting cell viability in another manner. Rather, P-Rex1-deficient PC12-S1PR1 cells were accumulated in G1 phase. Hence, P-Rex1 appears to regulate the proliferation of neuronal cells through effects on cell-cycle progression. The underlying mechanisms require further investigation, particularly in view of our finding that constitutive levels of Erk activity were unaffected by the P-Rex1 deficiency.

In conclusion, our study identified novel functional roles of the Rac-GEF P-Rex1 in neuronal GPCR signalling through Rac and Akt, in cAMP production, and in GPCR-dependent neuronal morphologies, as well as cell-cycle progression and proliferation. The underlying mechanisms of P-Rex1-dependent cAMP production and cell-cycle progression, in particular, warrant further investigation in the future.

## Data Availability

Data are contained within the article.
